# Infliximab: 12 years of experience

**DOI:** 10.1186/1478-6354-13-S1-S2

**Published:** 2011-05-25

**Authors:** Josef S Smolen, Paul Emery

**Affiliations:** 1 Division of Rheumatology, Department of Medicine III, Medical University of Vienna, Vienna, Austria; 2 2nd Department of Medicine, Hietzing Hospital, Waehringer Guertel 18-20, A-1090 Vienna, Austria; 3 Section of Musculoskeletal Disease, Leeds Institute of Molecular Medicine, University of Leeds; NIHR Leeds Musculoskeletal Biomedical Research Unit, Leeds Teaching Hospitals Trust, Leeds, Chapel Town Road, Leeds LS7 4RX, UK

## Abstract

Rheumatoid arthritis (RA), ankylosing spondylitis (AS) and psoriatic arthritis (PsA) are immune-mediated conditions that share an inflammatory mechanism fuelled by excessive cytokines, particularly TNF. Control of inflammation and rapid suppression of cytokines are important in treating these diseases. With this understanding and the corresponding advent of TNF inhibitors, RA patients, AS patients and PsA patients have found more choices than ever before and have greater hope of sustained relief. As a widely used TNF inhibitor, infliximab has a deep and established record of efficacy and safety data. Extensive evidence - from randomised controlled clinical trials, large registries and postmarketing surveillance studies - shows that infliximab effectively treats the signs and symptoms, provides rapid and prolonged suppression of inflammation, prevents radiologically observable disease progression and offers an acceptable safety profile in RA, AS and PsA. In very recent studies, investigators have observed drug-free remission in some patients. Additionally, infliximab may interfere with rapidly progressing disease in RA by early addition to methotrexate in patients with signs of an aggressive course. Finally, infliximab has been shown to reduce PsA clinical manifestations such as nail involvement. With our current understanding, substantial data and increasing confidence regarding use in practice, infliximab can be considered a well-known drug in our continued campaign against inflammatory rheumatic diseases.

## Insights into mechanisms

Rheumatoid arthritis (RA), ankylosing spondylitis (AS) and psoriatic arthritis (PsA) are all associated with a probably distinct immune-mediated pathogenesis that is central to the pathophysiology of each disease but ultimately leads to a chronic inflammatory response as a final common pathway. This fundamental inflammatory response is characterised by an overproduction of pro inflammatory cytokines, particularly TNF, IL-1 and IL-6 [1].

TNF is a dominant proinflammatory cytokine in RA, AS and PsA. The cytokine has both a direct effect and an indirect effect on the inflammatory events in these conditions [2-4]. TNF induces macrophages and other cells to secrete other proinflammatory cytokines (for example, IL-1, IL-6, IL-8), leads to T-cell activation and induces endothelial cells to express both adhesion molecules that increase T-cell infiltration and vascular growth factors that promote angiogenesis and keratinocyte proliferation. TNF is also involved in the differentiation and maturation of osteoclasts, the pivotal cells engaged in bone destruction in arthritis [5], and stimulates fibroblasts, osteoclasts and chondrocytes to release proteinases, which destroy articular cartilage and bone [1,3,6,7].

Typical inflammatory symptoms in RA include joint swelling and pain, systemic malaise and morning joint stiffness. As RA progresses, continued inflammation leads to permanent damage to the cartilage, bone, tendons and ligaments and, subsequently, to joint destruction and disability [1].

AS is primarily a disease of the axial skeleton that involves the sacroiliac joints and spine [8]. Inflammatory back pain with stiffness is the main clinical symptom [9]. Nonaxial involvement may include peripheral joint arthritis (most commonly of the knees), enthesitis and dactylitis [10,11]. Extra-articular manifestations are fairly common in AS patients [12-14] and can affect the eyes, gastrointestinal tract, lungs, heart and bones.

PsA is characterised by joint damage with associated pain and swelling. The disorder is similar to RA but with less severe symptoms. Nail abnormalities, psoriatic skin lesions, enthesitis and dactylitis are common in PsA [15]. Nail psoriasis is associated with a higher prevalence of joint involvement and a more progressive form of the disease [16,17]. The skin lesions usually manifest before arthritic symptoms [18].

## Targeting underlying inflammation

Disease control differs among RA, AS and PsA. In AS, nonsteroidal anti-inflammatory drugs can slow or inter fere with the associated radiographic changes [19] and are the cornerstone of symptom control, even though not all patients benefit [20]. In mild PsA, nonsteroidal anti-inflammatory drugs may also be sufficient to control symptoms and joint damage, since the disease’s propensity to destroy joints is frequently not high. In RA, however, nonbiologic (synthetic) disease-modifying anti-rheumatic drugs (DMARDs) (for example, sulphasalazine, methotrexate (MTX), leflunomide) are the mainstay of treatment, since they interfere not only with the signs and symptoms but also with progression of joint damage in many patients. These drugs also are effective in PsA; they have limited or no efficacy in axial AS, however, despite being effective in the other chronic inflammatory joint diseases and in peripheral arthritis of patients with AS [21,22].

Corticosteroids also have DMARD properties [23]. In RA, they are used in combination with synthetic DMARDs such as MTX (bridging therapy) to induce more rapid reduction of disease activity, and then are rapidly tapered. Corticosteroids are also used to treat oligoarthritis in PsA, although reactivation of psoriasis may occur upon steroid tapering. In AS, local corticosteroids can relieve site-specific inflammation, but systemic use in axial AS is not supported by available evidence [22]. Long-term use of these drugs is limited by their side-effect profile [24,25].

Although synthetic DMARDs are effective in many patients with RA and PsA, a considerable number require a different approach. Until the advent of biologic therapies, alternative medications did not exist and treatments often did not sufficiently control symptoms, joint damage and impairment of physical function. Consequently, confinement to a wheelchair and rapid loss of work ability were not infrequent. As understanding of the central inflammatory mechanism has improved and the role of TNF has been elucidated, however, therapies have shifted from mere interference with the magnitude of the inflammatory response to its abrogation and thus toward halting progression of joint damage and restoring physical function and work ability. Interference with the proinflammatory cytokine cascade using TNF inhibitors, but also interfering with other biological targets, may rapidly suppress and control inflammation and thereby prevent irreversible tissue damage and disability [26].

For a long time, only three TNF inhibitors were available for the treatment of RA, AS and PsA: adalimumab, etanercept and infliximab. Etanercept and infliximab were approved for the treatment of RA within a year of each other (1998 and 1999, respectively) in the United States and in the same year (2000) in Europe. Worldwide patient exposures for these three agents total almost 2 million patients [27-29].

Infliximab was the first biologic agent shown to be efficacious in RA, AS and PsA [30]. Later studies revealed that combination infliximab plus MTX tended to be superior to monotherapy [31], dramatically affected joint damage [32] and inhibited joint damage even in the absence of a clinical response, thus fostering the dissociation hypothesis (see Early rheumatoid arthritis, below) [33]. That these infliximab data were paradigmatic for the new class of TNF inhibitors has been shown in studies of other agents that fully confirmed the infliximab results [34-37]. An examination of the wealth of clinical data amassed over 12 years of experience with infliximab from its first licensing in Crohn’s disease (1998 in the United States) can thus tell us much about the state – and future – of TNF inhibitor therapy in RA, AS and PsA.

Whilst etanercept is not sufficiently efficacious in Crohn’s disease, the three TNF inhibitors appear to have similar efficacy in RA, PsA and AS. In the present review, we focus on infliximab as a prototypical example for these effects.

## Long-term infliximab use

The available data reveal that infliximab provides rapid and prolonged suppression of inflammation and inhibits progression of joint damage in many patients with RA and PsA [38-41]. In addition, TNF inhibition – such as that with infliximab – induces almost complete and sustained resolution of spinal inflammation in many patients with AS [42,43].

### Efficacy in rheumatoid arthritis

Infliximab has emerged as a highly effective treatment in both early and established RA [32,40,44,45].

#### Early rheumatoid arthritis

Efficacy in patients with early RA is critically important, since it is now understood that progression in inflam-mation severity and joint damage is slow in some patients and more rapid in others [46,47]. Rapidly progressing patients should be identified early in their disease course because they may benefit from more intensive therapy. The best predictors of rapidly progressing RA are currently the number of swollen joints, the presence of autoantibodies (high-titre rheumatoid factor and anti-citrullinated peptide antibodies) and elevated acute-phase response (as measured by the erythrocyte sedimentation rate (ESR) or C-reactive protein (CRP) level) [47-50].

**Figure 1:**
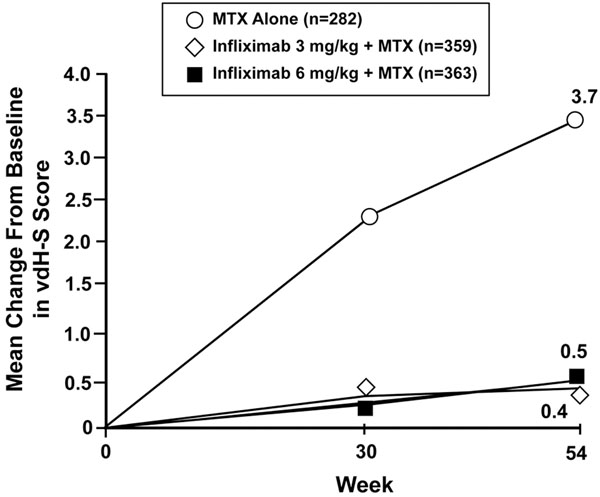
**Early rheumatoid arthritis: radiological progression.** Infliximab plus methotrexate (MTX) reduces progression of joint damage in rheumatoid arthritis compared with MTX alone (*P* < 0.001 at weeks 30 and 54) [45]. vdH-S, van der Heijde–Sharp.

In the ASPIRE trial, the efficacy of infliximab (3 or 6 mg/kg infusions at weeks 0, 2 and 6 and every 8 weeks thereafter) plus MTX (titrated up to 20 mg/week by week 4) was assessed in 1,004 MTX-naïve patients with early (≥3 months, ≤3 years), moderate-to-severe active RA over a 54-week period [45]. Infliximab plus MTX provided significantly greater clinical, radiological and functional benefits than MTX alone in patients with early RA. At week 54 there were no significant differences in clinical efficacy between the infliximab groups – but compared with MTX alone, the American College of Rheumatology (ACR)-N, ACR20, ACR50 and ACR70 response rates were significantly higher with infliximab. From baseline to week 54, the change in radiological progression was significantly less in patients receiving infliximab 3 mg/kg plus MTX and infliximab 6 mg/kg plus MTX than in those receiving MTX alone (van der Heijde–Sharp scores, 0.4 ± 5.8, 0.5 ± 5.6 and 3.7 ± 9.6, respectively; Figure 1). In addition, improvements in physical function (Health Assessment Questionnaire) were significantly greater in both infliximab treatment groups compared with the MTX-alone group [45].

Another report from the ASPIRE trial investigated the prognostic value of disease activity markers (laboratory, clinical and radiographic) in relation to progression of joint damage [50]. In patients receiving MTX alone, a higher swollen joint count, higher ESR and CRP levels and higher rheumatoid factor levels at baseline were significantly correlated with greater joint damage at week 54. This correlation was abrogated in patients treated with infliximab plus MTX because of the marked effects on joint damage irrespective of the underlying disease activity or auto antibody state. An additional analysis that adjusted for baseline demographic and other clinical characteristics still found an increased ESR and increased swollen joint counts to be significantly associated with greater joint damage at week 54 in the MTX-alone group. Neither of these markers, however, was predictive of greater joint damage in the infliximab-plus-MTX group. The Disease Activity Score in 28 joints (DAS28) was mostly high at baseline in all patients; decreases were seen after 12 weeks. At 14 weeks, patients in the MTX-alone group who had higher DAS28 scores showed greater progression of joint damage at week 54 than those in the group with lower scores. Again, no such correlation was noted in the infliximab-plus-MTX group.

Radiographic progression, as determined by van der Heijde–Sharp scores, was also greatest in the portion of the MTX-only group that had the highest baseline CRP level and ESR: at 54 weeks, the score changed by 1.81 points (± 7.27) in patients with normal CRP levels and ESR, and by 4.71 points (± 10.69) in patients with high CRP levels (≥0.8 mg/dl) and high ESR (>15 to 20 mm/ hour) [50]. In the infliximab-plus-MTX group, however, the baseline CRP level and ESR had little association with radiographic progression; infliximab plus MTX inhibited radiographic progression regardless of baseline disease activity or joint damage. In fact, all anti-TNF agents, when combined with MTX, are very effective in preventing radiological damage.

**Figure 2:**
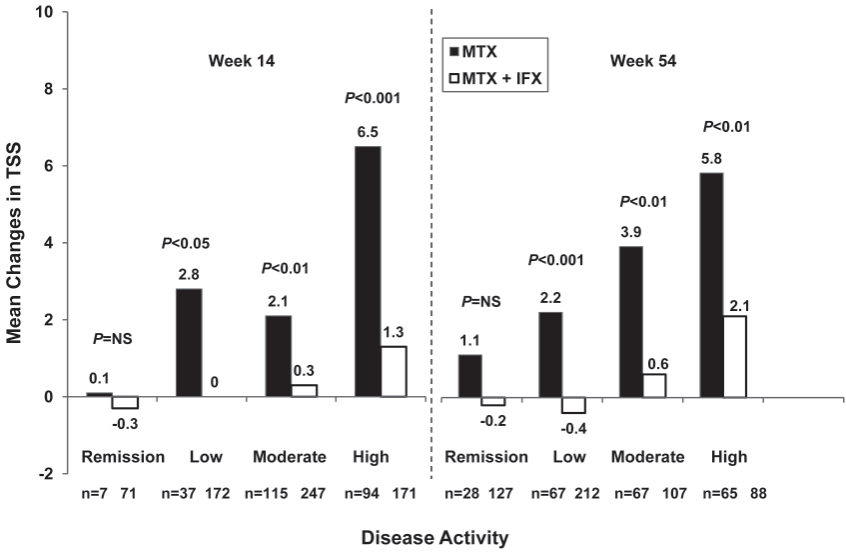
**Early rheumatoid arthritis: disease activity.** Changes in total Sharp score (TSS) by disease activity states, as classified by the simplified disease activity index. IFX, infliximab; MTX, methotrexate; NS, not significant. Modified from [51].

Importantly, only patients attaining stringent remission by the criteria of the simplified disease activity index at week 14 did not progress radiologically irrespective of therapy; while those on MTX, when attaining low or higher categories of disease activity at week 14, progressed with increasing disease activity state. In contrast, infliximab plus MTX halted radiologic progression even if patients had achieved low or moderate disease activity at week 14 [51], confirming previous notions that this treatment dissociates the traditional link between inflammation and destruction [33]. According to this dissociation hypothesis, treatment reduces the impact of inflammation on destruction to the extent that some progression of damage is seen only in patients with very high levels of unsuppressed inflammation (Figure 2). Whether stringent remission was achieved at 3 months or 1 year, there was an almost linear increase in progression of joint damage with MTX, reaching approximately 6 radio-graphic score points with high disease activity (Figure 2). The radiographic progression was not only fully or mostly abrogated with infliximab plus MTX in remission, but also in low and even moderate disease activity [51]. Nevertheless, even with combination therapy there was a link between disease activity and progression of joint damage, although the slope was dramatically diverted. Therefore, although there is still a connection between inflammation and destruction, TNF-inhibitor-plus-MTX treatment reduces the impact of inflammation on destruction to the extent that progressive damage is seen only in cases with high levels of unsuppressed inflammation – but even then to a lesser degree than upon treatment with MTX alone.

#### Rapidly progressing disease in rheumatoid arthritis

Although the efficacy of MTX is appreciable, patients with rapidly progressing disease (RPD) may obtain additional benefit from more intensive therapy. The CRP level and ESR may serve as predictors of future joint damage in patients with early RA who are treated with MTX monotherapy and may allow potentially optimal management with the earlier addition of a TNF inhibitor. Although few studies have been performed in patients with RPD despite MTX therapy, analyses of subsets of these patients have demonstrated improved long-term benefits with the early addition of infliximab [33,52,53]. Infliximab has been evaluated in this regard in both early and long-term disease with similar results. Likewise, starting these patients on etanercept monotherapy or adalimumab plus MTX has shown similar efficacy [54,55]. The GUEPARD trial, however, showed that rapid addition of a TNF inhibitor to a DMARD – if the latter has not been sufficiently effective within 3 to 6 months – provides clear-cut benefit similar to that derived from starting with combination anti-TNF and DMARD therapy [56].

**Figure 3:**
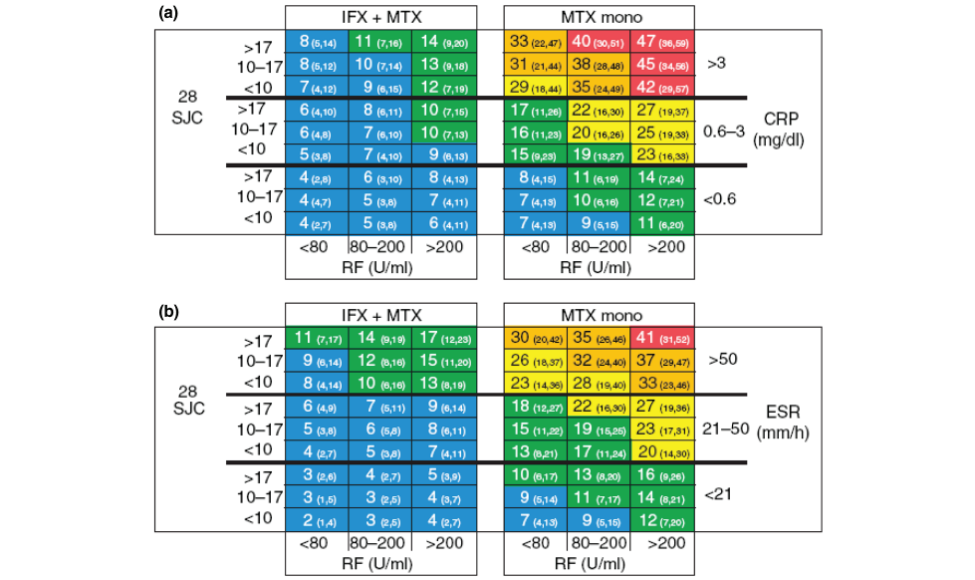
**Rapidly progressing disease in rheumatoid arthritis.** Matrix risk model for the probability of rapid radiographic progression (RRP) in 1 year, including all selected baseline risk factors, except **(a)** erythrocyte sedimentation rate (ESR) or **(b)** C-reactive protein (CRP), generated from the ASPIRE early rheumatoid arthritis dataset. Numbers in each cell represent the percentage (95% confidence interval) of patients who had RRP out of all patients who have the baseline characteristics and receive the initiated treatment as indicated. Predicted probability of RRP: blue, 0 to 9%; green, 10 to 19%; yellow, 20 to 29%; orange, 30 to 39%; red, 40 to 100%. A higher percentage indicates more severe radiographic progression of joint damage. IFX, infliximab; mono, monotherapy; MTX, methotrexate; RF, rheumatoid factor; SJC, swollen joint count. Reprinted with permission from [47].

The prediction of RPD in patients with RA represents an intriguing challenge for tailoring biologic therapy and an exciting development in the field. Two pilot risk models for predicting RPD in RA patients were recently proposed [47]. ASPIRE data were used to define RPD and to identify baseline risk factors; in line with previous data [50], these risk factors were swollen joint counts, rheumatoid factor levels, CRP levels and the ESR. The results were then combined with initiated treatments and arranged in matrices that allow prediction of risk in 1 year (Figure 3). One model incorporated all risk factors except the CRP level, and the other model incorporated all risk factors except the ESR, to enable interchangeable use depending on clinical availability. Both models identified subpopulations of RA patients at higher predicted risk of RPD, particularly those who were MTX-naïve with early disease. Additional development plus testing of the models in other RA populations is needed (and currently in progress) to produce a single tool that would be practical and validated for use in everyday practice.

Very early transient treatment with infliximab has been shown to be effective in early, poor-prognosis RA. In a randomised, double-blind study, 20 previously untreated patients with early (<12 months), poor-prognosis RA (defined by the Persistent Inflammatory Symmetrical Arthritis scoring system) were randomised to receive infliximab plus MTX (3 mg/kg) or placebo plus MTX for 12 months [53]. After 1 year of treatment, magnetic resonance imaging (MRI) evidence of synovitis and joint damage was reduced (with significantly fewer new erosions) in infliximab-treated patients compared with MTX-alone patients. Significantly more patients receiving infliximab plus MTX than those receiving MTX alone were ACR50 responders (78% and 40%, respectively) and ACR70 responders (67% and 30%, respectively). Furthermore, greater improvements in physical function (Health Assessment Questionnaire) and quality of life (QoL) (determined by the Rheumatoid Arthritis Quality of Life questionnaire) were seen throughout the 12 months of treatment in the infliximab-plus-MTX group. Treatment was stopped after 1 year, and the patients were then followed for another 12 months. One year post infliximab-plus-MTX therapy, clinical response was sustained in 70% of the patients in this group, with a median DAS28 of 2.05. Significant improvements in function and QoL were also sustained [53].

The BeST study, a randomised trial that assessed four different treatment strategies in 508 patients with recent-onset RA, showed similar results. Over year 1, patients receiving initial combination therapy with tapered high-dose prednisone plus MTX plus sulphasalazine (group 3, 133 patients) or infliximab plus MTX (group 4, 128 patients) had more rapid functional improvement and less progression of radiographic joint damage than patients treated with sequential monotherapy (group 1, 126 patients) or step-up combination therapy (group 2, 121 patients), and the differences at most time points were significant [57]. The BeST study also demonstrated that clinical and functional benefits of infliximab plus MTX were maintained over 4 years [58,59]. In addition, the study provided important information about remission in RA. After 2 years of infliximab combination therapy, 67 out of 120 patients in group 4 (56%) were able to discontinue treatment, and 40 out of the 67 (33% of the total group 4 population) achieved clinical remission [60]. Moreover, significantly more patients in this group (16%) maintained clinical remission off infliximab than in groups 2 and 3, who received infliximab later in the course of their treatment (6% and 7%, respectively; *P* <0.05 for all) [58]. (The difference between groups 1 and 4 was not significant.) After 3 years of combination therapy, 31% of patients in group 3 and 48% of patients in group 4 were able to taper their medication to DMARD monotherapy or no DMARD. Finally, at year 4 – when 61 out of 120 patients (51%) were off infliximab – 20 out of the 61 (17%) were in complete remission, which lasted on average 1 year [59].

Six-year BeST data were presented at the October 2009 ACR scientific meeting. Of the original 508-patient study population, 99 patients (19%) withdrew over 6 years. Of the remaining 409 patients, 51% were in clinical remission at 6 years, and 17% (36 patients) of those in remission were in prolonged drug-free remission [61].

#### Established rheumatoid arthritis

Infliximab has also demonstrated efficacy in patients with established RA. The ATTRACT study evaluated the efficacy of infliximab in 428 patients with active RA of 7.2-year to 9-year duration, despite 3 months or more of MTX therapy [32,40,44]. Patients received 3 mg/kg or 10 mg/kg infusions of infliximab at weeks 0, 2 and 6 and then at 4 or 8 weeks thereafter in combination with MTX. This randomised, double-blind, placebo-controlled phase III study showed that infliximab plus MTX is effective in controlling the signs and symptoms of established RA. After 30 weeks of assessment, 51.8% of patients receiving any dose of infliximab plus MTX demon strated a clinical response (≥20% improvement from baseline using ACR assessment criteria (ACR20)) compared with only 17% of patients receiving placebo plus MTX [32,44]. Furthermore, approximately 30% of infliximab-plus-MTX patients achieved a 50% improvement from baseline compared with only 5% of placebo-plus-MTX patients [44].

**Figure 4:**
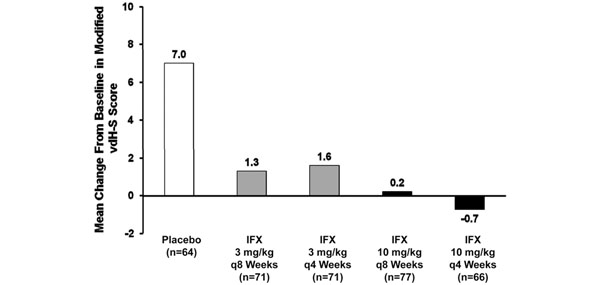
**Established rheumatoid arthritis: progression of structural joint damage.** Infliximab (IFX) plus methotrexate (MTX) significantly reduces progression of structural joint damage compared with MTX alone, after 1 year of treatment. All patients received concomitant MTX [32]. *P* < 0.001 for all doses and courses. vdH-S, van der Heijde–Sharp.

**Figure 5:**
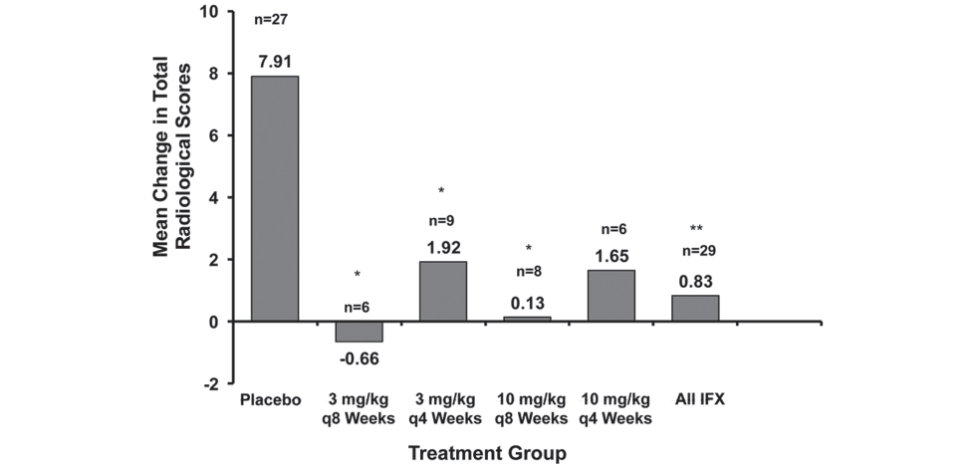
**Established rheumatoid arthritis: inflammation and joint destruction.** Mean change from baseline to week 54 in modified van der Heijde–Sharp score among patients who remained clinical nonresponders from week 2 through week 54, by treatment group. Corresponding median changes in the methotrexate (MTX)-plus-placebo-treated group (placebo) and the groups receiving infliximab (IFX) 3 mg/kg every 8 weeks plus MTX, IFX 3 mg/kg every 4 weeks plus MTX, IFX 10 mg/kg every 8 weeks plus MTX, and IFX 10 mg/kg every 4 weeks plus MTX, as well as all IFX-plus-MTX groups were 3.50, 0.27, –0.50, –0.25, 1.25 and 0.00, respectively. *P <0.05, **P <0.01 versus MTX-plus-placebo-treated patients. Dosage and frequency data (4 weeks, 8 weeks) refer to infliximab treatment. Modified with permission from [33].

The ATTRACT study also showed that infliximab plus MTX significantly reduced progression of structural joint damage in RA compared with MTX alone [32]. After 1 year of treatment, infliximab plus MTX prevented the progressive joint damage associated with inflammation and resulted in a significant reduction in progression of radiological changes, using the modified van der Heijde– Sharp score, in a significant proportion of patients compared with placebo plus MTX (van der Heijde–Sharp scores, 1.63 and 6.95, respectively; Figure 4). Interestingly, the ATTRACT study also assessed the relationship between inflammation and joint destruction in patients not sufficiently responding clinically to infliximab plus MTX (ACR20 nonresponders), and found that infliximab plus MTX still provided inhibition of structural damage compared with placebo (plus insufficiently effective MTX) [32]. These results suggest that these two disease measures, which are usually tightly linked, are dissociated under this treatment (Figure 5) [33]. This suggestion was confirmed when it was shown that joint damage was retarded even in patients who had no improvement in disease activity measures [33], and similar findings were made in early RA [51], as discussed above (see Figure 2).

Studies of long-term infliximab therapy have demonstrated that the positive effects on joint damage are sustained. For example, at the end of ATTRACT year 2, the data showed significant improvements in clinical response and inhibition of progressive joint damage with infliximab plus MTX compared with placebo (plus insufficiently effective MTX) [40]. Indeed, patients receiving infliximab plus MTX not only continued to have good clinical responses and inhibition of progressive joint damage during that 2-year period, but also experienced significant improvements in physical function (as determined by the self-administered Health Assessment Questionnaire) and health-related QoL (as determined by the Short-form 36 Health Survey) com pared with patients receiving placebo (plus insufficiently effective MTX) [40].

Another study of 511 patients with longstanding, refractory RA found that long-term maintenance therapy with infliximab continues to reduce disease activity [62]. The researchers also examined 4-year compliance rates and found that a majority of patients continued treatment. Infliximab was well tolerated, and 61.6% of patients were still receiving this treatment at the 4-year point [62]. The main reasons for discontinuing therapy were lack of efficacy (13.6%) and safety issues (16.9%). This study is in line with smaller studies demonstrating 3-year infliximab continuation rates of 58 to 75% [63-68].

### Efficacy in ankylosing spondylitis

**Figure 6:**
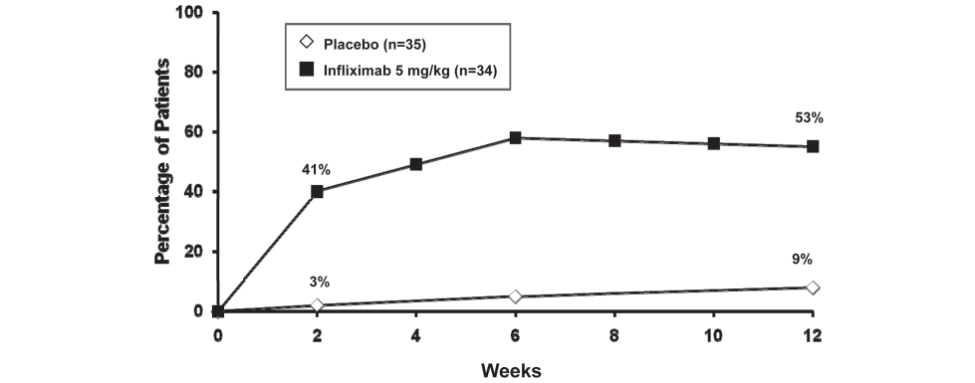
**Ankylosing spondylitis: disease activity.** Infliximab rapidly reduces disease activity compared with placebo (*n* = 70 patients). Percentage of patients with improvement ≥50% in the Bath Ankylosing Spondylitis Disease Activity Index. *P* <0.0001 from 2 weeks onwards. Modified with permission from [71].

Infliximab induces a rapid reduction in disease activity in patients with AS. The TNF-inhibitor affects the underlying inflammation of both articular and extra-articular manifestations of AS [2,12,69,70]. Significant efficacy compared with placebo was first reported by Braun and colleagues in a randomised, double-blind study of 69 patients with active AS [71]. After 12 weeks of treatment, 53% of patients receiving infliximab (5 mg/kg) had ≥50% reduction in disease activity, as measured by the Bath Ankylosing Spondylitis Disease Activity Index (BASDAI), compared with 9% of patients receiving placebo (Figure 6). The physical function, as measured by the Bath Ankylosing Spondylitis Functional Index, and QoL (Short-form 36 Health Survey) also significantly improved in infliximab-treated patients compared with placebo-treated patients (both *P* <0.0001).

**Figure 7:**
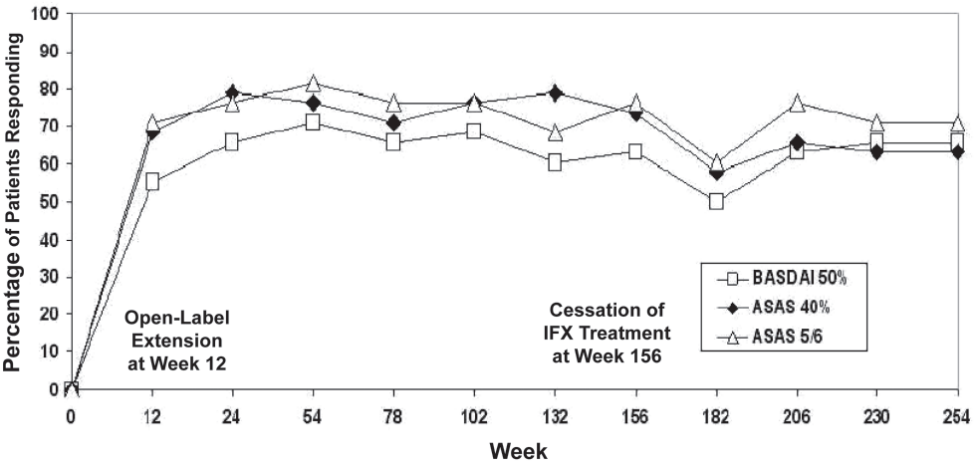
**Ankylosing spondylitis: improvement in disease activity.** Infliximab sustains improvement in disease activity over 5 years. At 156 weeks, *n* = 43 patients; at 254 weeks, *n* = 38. ASAS, Assessment of SpondyloArthritis International Society; BASDAI, Bath Ankylosing Spondylitis Disease Activity Index; IFX, infliximab. Modified with permission from [74].

After the 12-week, placebo-controlled phase of this study, all patients entered a 3-year open-label extension. Sixty-two per cent (43 out of 69 patients) completed 3 years of infliximab treatment and then discontinued to allow assessment of the time to flare. Most patients relapsed within 18 to 24 weeks, and 42 out of 43 patients restarted infliximab, with most regaining efficacy. Good clinical response was sustained for 5 years, with 63%, 58%, 61% and 63% of patients achieving at least a 50% reduction in disease activity (BASDAI) from baseline after 1, 2, 3 and 5 years of treatment, respectively (Figure 7) [72-74]. The Assessment of the Spondylo-Arthritis International Society (ASAS) ASAS40 response was seen in 75% and 63% of patients at the end of years 3 and 5, respectively. Similarly, an ASAS5/6 response was achieved in 76% and 71% of patients at the end of years 3 and 5, respectively (see Figure 7). Low disease activity (BASDAI value <3 units) was attained in 57.9% of patients after 5 years; the mean BASDAI was 2.5 ± 1.9 (baseline, 6.4; at 3 years, 2.5) [74]. Partial clinical remission (score ≤2 in each of the four ASAS domains) was reached by 37% and 34% of patients at the end of years 3 and 5, respectively [74]. The time to flare during discontinuation suggested that continuous therapy is necessary to achieve a lasting effect in patients with severe, active AS.

**Figure 8:**
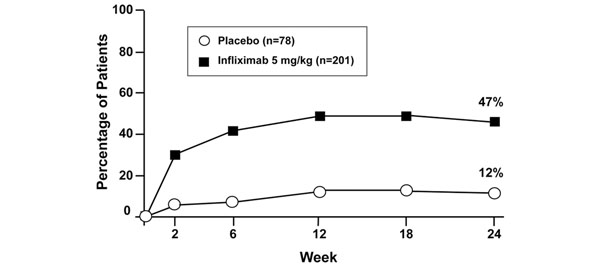
**Ankylosing spondylitis: rapid clinical response.** Infliximab rapidly improves ASAS40 compared with placebo (*n* = 279 patients). *P* <0.001 from 2 weeks onwards. ASAS, Assessment of SpondyloArthritis International Society. Modified with permission from [75].

The ASSERT trial also provided evidence for the efficacy and safety of infliximab (5 mg/kg) in patients with AS [75]. In this randomised, placebo-controlled study of 279 patients, the clinical response was rapid, as early as 2 weeks, and was sustained over 24 weeks, with 61.2% of infliximab patients achieving ASAS20 compared with 19.2% of placebo patients at week 24 (*P* <0.001). In addition, 47% of patients in the infliximab group were ASAS40 responders compared with 12% of patients in the placebo group at week 24 (Figure 8). Significant improvements in the BASDAI and the Bath Ankylosing Spondylitis Functional Index were also seen in the patients receiving infliximab.

**Figure 9:**
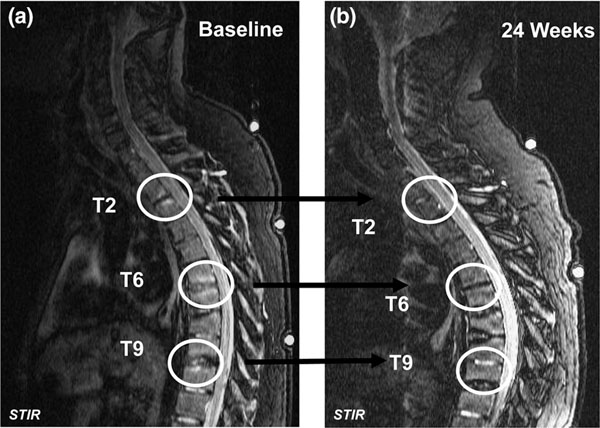
**Ankylosing spondylitis: spinal inflammation.** Infliximab completely resolves spinal inflammation in most patients: **(a)** before-treatment and **(b)** after-treatment gadolinium-enhanced T1 images. STIR, short-tau inversion recovery. Reproduced with permission from [42].

Infliximab induced a pronounced reduction in spinal inflammation in MRI examinations of patients in the ASSERT study. The MRI activity score improved significantly more in infliximab-treated patients (mean, 5.02; median, 2.72) compared with placebo patients (mean, 0.60; median, 0.0) from baseline to week 24 [42]. Most infliximab-treated patients achieved complete resolution of spinal inflammation (Figure 9).

The reduction in spinal inflammation with infliximab was sustained over the long term. At week 24 of the ASSERT study, placebo patients crossed over to receive infliximab (5 mg/kg) as part of an open-label extension [43]. Short-tau inversion recovery MRI images were taken at baseline and at weeks 24 and 102. Patients in the infliximab group showed improvement in the Ankylosing Spondylitis MRI Spinal Score for Activity at week 24 (mean, –4.4; median, –2.00) compared with no change in the placebo group (mean, 0.38; median, 0.25), and this improvement was sustained through 102 weeks [43]. Patients in the placebo group improved after crossing over to receive infliximab at week 24, and subsequently achieved similar levels of spinal inflammation reduction by week 102 as patients receiving infliximab from the start. Interestingly, however, and contrasting with results in RA, infliximab does not appear to halt progression of radiographic changes; likewise, structural changes also progressed on etanercept treatment, contrasting the clinical effects [75,76].

In another study, 40 patients in whom early sacroiliitis had been determined by MRI were randomised in a double-blind manner to infliximab 5 mg/kg or placebo at 0, 2, 6 and 12 weeks. Both MRI and clinical assessment at 16 weeks showed significantly reduced disease activity. For example, significantly more lesions resolved in the infliximab group (*P* <0.001), while significantly more new lesions developed in the placebo group (*P* = 0.004) [77].

Infliximab was also found to mitigate extra-articular manifestations of AS, which can reduce QoL and signal worse outcomes. For example, patients with AS have a 20 to 30% risk of uveitis [78], and a meta-analysis showed that infliximab significantly reduced the incidence of uveitis compared with placebo (*P* = 0.005) [70]. Sub-clinical inflammation of the gut is present in up to 60% of AS patients, and this inflammation can evolve into fullblown inflammatory bowel disease [79]. Another meta-analysis showed that infliximab significantly reduced incidence rates of flares or new-onset inflammatory bowel disease compared with etanercept (*P* = 0.001) and adalimumab (*P* = 0.02) [80]. Similarly, a subanalysis of the ASSERT trial’s 24-week phase demonstrated significant increases in mean spinal bone density in AS patients treated with infliximab compared with placebo (*P* <0.001) [81]. The effects on vertebral fracture, however, are not yet known.

### Efficacy in psoriatic arthritis

Infliximab is effective in treating various aspects of PsA, including joint symptoms and extra-articular manifestations such as dactylitis, enthesitis and nail disease, as well as psoriatic skin involvement. The efficacy of infliximab in PsA was assessed in the IMPACT 1 and IMPACT 2 studies [82,83]. These studies were similar in design, with a 16-week to 24-week, randomised, placebo-controlled phase, after which all patients received infliximab for up to 1 year. Both studies measured articular and composite disease assessment, skin symptoms, enthesitis, dactylitis and QoL. Enrolled patients had active PsA that was unresponsive to at least one DMARD.

**Figure 10:**
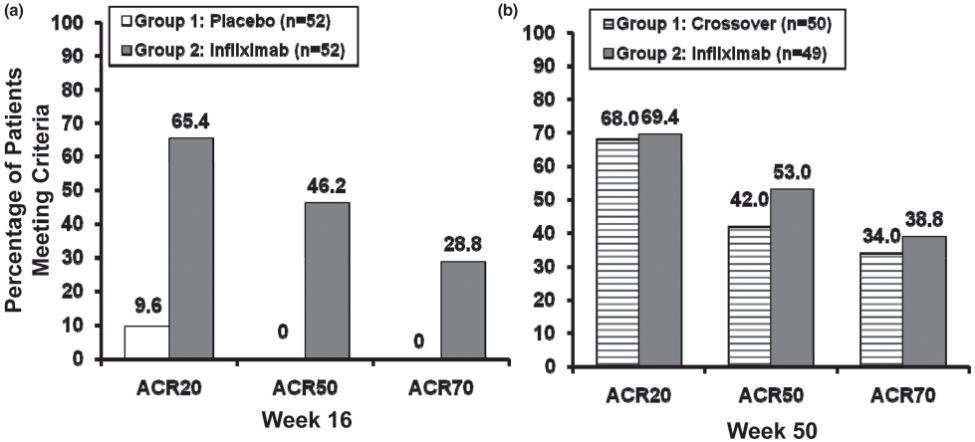
**Psoriatic arthritis: improvement of joint symptoms.** Infliximab significantly improves joint symptoms compared with placebo (*P* <0.001 at week 16) in the IMPACT 1 study (*n* = 104 patients) [82]. **(a)** Prior to crossover. **(b)** Open-label extension, up to 54 weeks. ACR, American College of Radiology.

Significant improvements were observed in the signs and symptoms of articular disease. In the IMPACT 1 study (*n* = 104), 65.4% of patients treated with infliximab were ACR20 responders at week 16 compared with only 9.6% of placebo patients (Figure 10a) [82]. Furthermore, 46.2% and 28.8% of infliximab-treated patients were ACR50 and ACR70 responders, respectively, compared with none of the placebo patients at week 16. Similar improvements were seen in the IMPACT 2 study (*n* = 200): 58% of infliximab-treated patients and 11% of placebo patients achieved an ACR20 response at week 14 (*P* <0.001) [83]. Forty-one per cent and 27% of patients in the infliximab group were ACR50 and ACR70 responders, respectively, compared with 4% and 2% of placebo patients, respectively, at week 24. The improvement in joint symptoms was sustained throughout both studies (up to 54 weeks) [82,84]. In IMPACT 1, for example, the proportion of ACR20 responders in the group of placebo patients who crossed over to infliximab treatment was similar to the proportion of ACR20 responders in the group of patients who received infliximab from day 1: 68% and 69%, respectively (Figure 10b) [82].

Infliximab also inhibits the radiological progression of joint damage in PsA [41,85]. During the placebo-controlled phase (weeks 1 to 24) of the IMPACT 2 study, radiographs of the hands and feet showed significantly less progression of structural damage in infliximab patients compared with placebo patients (mean change from baseline in modified van der Heijde–Sharp score, –0.70 and 0.82, respectively) [41]. The mean annual progression rate at baseline was equivalent to 5.8 modified van der Heijde–Sharp points/year for the overall study population, but the projected rate for the overall population post infliximab was –1.79 [85]. In fact, 84.3% of the total patient population did not have radiographic progression after a year of infliximab treatment [85].

Infliximab also improved physical function in PsA regard less of baseline radiographic damage. After 54 weeks of treatment, the percentage improvement in the Health Assessment Questionnaire was strikingly better than at baseline in both treatment groups [84]. Importantly, those patients with less radiological damage regained function more quickly, suggesting that therapeutic intervention early in the disease course may limit the amount of joint damage.

**Figure 11:**
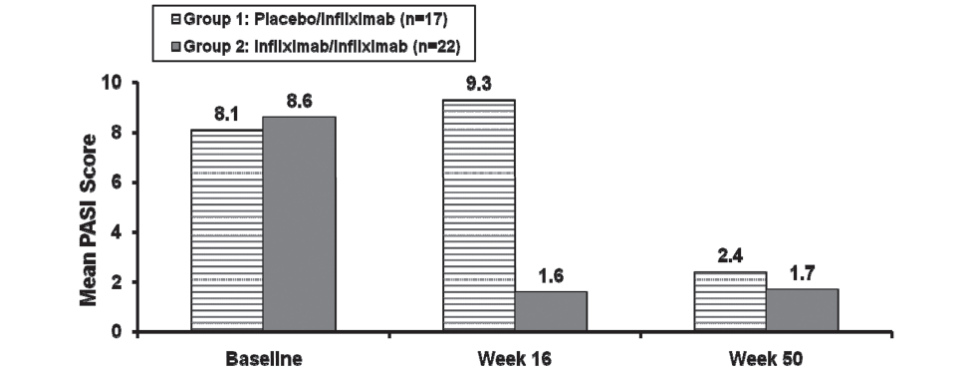
**Psoriatic arthritis: improvement of skin symptoms.** Infliximab improves skin symptoms in patients with psoriatic arthritis at week 16 (*P* < 0.001) and after crossover in the IMPACT 1 study. PASI, Psoriasis Area and Severity Index. Modified from [82].

Additionally, infliximab was effective in treating psoriatic skin symptoms. In the IMPACT 1 study, infliximab-treated patients with a Psoriasis Area and Severity Index (PASI) score ≥2.5 at baseline had a mean improvement from baseline in PASI score of 86% compared with a 12% deterioration in placebo patients (*P* <0.001) [82]. Of these, 68% of infliximab-treated patients achieved an improvement in PASI score ≥75% compared with none of the placebo patients (*P* <0.001). Improvements were maintained over 50 weeks (Figure 11). Similar findings were observed in the IMPACT 2 study; 64% of patients with skin involvement treated with infliximab achieved an improvement in PASI ≥75% compared with 2% of placebo patients (*P* <0.001) [83]. Interestingly, another study observed a correlation between skin response and improvement in joint symptoms in PsA patients treated with infliximab. Patients with a good skin response had a greater joint response than those with no skin response [86].

The IMPACT 2 study also evaluated the effect of infliximab on the incidence of other typical features of PsA. Dactylitis was less frequent in infliximab-treated patients than placebo patients at both week 14 (18% vs. 30%, *P* = 0.025) and week 24 (12% vs. 34%, *P* <0.001). Enthesopathy was also less frequent in infliximab-treated patients than in placebo patients at both week 14 (22% vs. 34%, *P* = 0.016) and week 24 (20% vs. 37%, *P* = 0.002) [83].

The EXPRESS trial was the first large, controlled, phase III clinical study to use the Nail Psoriasis Severity Index tool in patients with psoriasis [87]. Of the 378 patients randomised, 114 (30.2%) had a history of PsA. Among the 373 patients evaluated for nail disease, it was found to be present at baseline in 87.5% of patients (98 out of 112) with a history of PsA and in 79.3% of patients without a history of PsA. At week 24, the mean percentage improve ments in nail bed scores in patients receiving infliximab versus those receiving placebo were 69.2% and 18.4%, respectively; the percentage improvements in nail matrix scores were 52.9% and –1.9%, respectively (*P* <0.001). Significant and comparable degrees of improvement were observed, regardless of baseline history of PsA. The Nail Psoriasis Severity Index values persevered in both groups at weeks 38 and 50 (placebo crossover to infliximab occurred at week 24), also regardless of PsA history.

The recently concluded RESPOND trial investigated an aggressive strategy in early, severe polyarticular PsA [88]. This study compared the efficacy and safety of infliximab 5 mg/kg plus MTX with MTX alone in MTX-naïve subjects who had an inadequate response to steroids and nonsteroidal anti-inflammatory drug therapy. The primary end point (ACR20 at week 16) was achieved in 44 out of 51 patients (86.3%) in the infliximab-plus-MTX group compared with 32 out of 48 patients (66.7%) in the MTX-alone group (*P* = 0.021). The ACR50 and ACR70 response rates at week 16 were also significantly greater in the infliximab-plus-MTX group, with 37 out of 51 patients (72.5%) achieving ACR50 (compared with 19 out of 48 patients (39.6%) in the MTX-alone group; *P* = 0.0009) and 25 out of 51 patients (49%) achieving ACR70 (compared with nine out of 48 patients (18.8%) in the MTX-alone group; *P* = 0.0015). Overall, patients receiving infliximab plus MTX showed more profound levels of disease suppression, as illustrated by DAS28 remission rates, an absence of swollen or tender joints, a normal CRP level and PASI 90 responses.

### Safety considerations

With 12 years of clinical use and the availability of national disease registries, the safety profile of TNF inhibitors is well characterised. Serious adverse events (SAEs) with infliximab include: the development of viral, fungal or bacterial infectious diseases (for example, tuberculosis (TB), listeriosis, sepsis, opportunistic infections due to *Cryptococcu*s, *Aspergillus* and *Pneumocystis*); reactivation of hepatitis B virus; hepatobiliary disorders (for example, worsening of hepatitis C, chole cystitis and cholelithiasis, very rare jaundice and non-infectious hepatitis); allergic/ infusion-related reactions (for example, anaphylaxis); malignancies (for example, lymphoma, nonmelanoma skin cancer); autoantibody formation (for example, lupus-like syndrome); haematological reactions (for example, pancytopaenia, aplastic anaemia); neurological disorders (for example, optic neuritis, seizure, demyelinating disorders such as multiple sclerosis); and worsening of congestive heart failure [89]. In general, as with efficacy, the safety aspects of TNF inhibitors are similar [90,91], and registries compile data on all of the biologics. The risks are thus recognised and are increasingly understood.

In an assessment of safety profiles for DMARDs and biologic agents in more than 10,000 patients with RA, no unexpected safety signals and no trends of concern were noted compared with data seen during earlier trials and in the early days of TNF-inhibiting therapies [92]. The assessment was based on the RADIUS trial, and also showed that rates of SAEs and and serious infections across multiple therapies were comparable with the rates observed with MTX treatment. Similar conclusions were drawn from an observational cohort of the Consortium of Rheumatology Researchers of North America registry, which included 18,305 RA patients [93]. There was no significant increase in the adjusted risk for overall infections associated with anti-TNF therapy compared with MTX, and the infection-related safety profiles of the various biologic agents appeared to be similar.

Serious infection rates were calculated in a prospective, observational study of 7,664 patients treated with TNF inhibitors and 1,354 patients treated with DMARDs from the British Society for Rheumatology Biologics Register [94]. All patients had severe RA. The crude rates of serious infections were found to be similar among TNF inhibitors: 51.3 events/1,000 person-years for etanercept, 55.2 events/1,000 person-years for infliximab and 51.9 events/1,000 person-years for adalimumab. During the study period, however, there were 525 serious infections in the TNF-inhibitor cohort and 56 in the DMARD cohort (9,868 and 1,352 person-years of follow-up, respectively). The incidence rate ratio, adjusted for baseline risk, for the TNF-inhibitor cohort compared with the DMARD cohort was 1.03 (95% confidence interval, 0.68 to 1.57), suggesting similar risk levels between the two treatment groups. The types of serious infections were different between the groups, however, with 19 serious bacterial intracellular infections occurring exclusively in patients in the TNF-inhibitor cohort. After adjustment for baseline risk, anti-TNF therapy was not associated with an increased risk of overall serious infections compared with DMARD treatment in patients with active RA [94]. Nevertheless, the data did show an increased risk of TB infection in patients treated with infliximab and other anti-TNF therapies, although this risk might be lower with etanercept [95].

A large randomised, placebo-controlled trial assessed the risk of serious infections following infliximab-plus-MTX therapy in patients with active RA [96]. The risk of serious infections in patients receiving infliximab 3 mg/kg plus MTX was similar to that in patients receiving MTX monotherapy. Furthermore, most infections reported in clinical trials of TNF inhibitors were minor and were treated with either outpatient antibiotic therapy and/or temporary withdrawal of the drug [97].

A prospective cohort study of the German RA registry RABBIT compared the rates of infections in patients treated with the biologic agents infliximab, etanercept and anakinra with the rates of infections in patients receiving conventional DMARDs. Among patients receiving infliximab, 21% experienced a serious infection compared with 6% of control patients. In addition, the incidence of adverse events in general was 3.3 to 4.1 times higher in patients receiving biologic agents than in the control group [98].

The immunosuppressive activity of TNF inhibitors conveys a theoretical risk of malignancy development. Postmarketing surveillance, however, reported lymphoma rates (mostly non-Hodgkin’s lymphoma) of between 0.01 and 0.03 events/100 patient-years in patients receiving TNF inhibitors [99]. The expected rate was 0.07 events/100 patient-years in a normal population aged 65 years. Further more, the potential rate of lymphoma was com plicated by the association of some immune-mediated diseases, especially RA, with an inherent lymphoma risk [100]. Currently, no clear association between infliximab and lymphoma has been established [101]. Cumulatively, 565 cases of lymphoma development have been reported among more than 1 million patients since the launch of infliximab. The cumulative rate for lymphoma per 100 patient-years since first exposure is 0.017 [101]. Although a definitive conclusion regarding lymphoma risk with TNF inhibitors in general – and infliximab in particular – cannot be reached at present, postmarketing pharmaco vigilance continues to track lymphoma incidence.

Injection site or infusion reactions occur with all TNF inhibitors – but because infliximab is a human-plus-mouse (that is, chimeric) antibody, anaphylaxis is possible. Anaphylactic reactions are uncommon in patients receiving infliximab [89]. In clinical trials, 5,706 patients received 36,485 infliximab infusions, for a mean of 6.4 infusions/patient, and 3,722 patients received 15,379 placebo infusions, for a mean of 4.1 infusions/ patient. Overall, the frequency of infusion reactions was 4% for infliximab compared with 1.6% for placebo. The majority of infusion reactions were mild to moderate (for example, nausea, headache, sweating, flushing). The rate of serious infusion reactions was 0.2% for infliximab and zero for placebo [102,103]. Immunogenicity can also arise (incidence, 9 to 17%). Although the effect of immuno genicity on efficacy is unclear, patients who develop immunogenicity may be at higher risk for infusion reactions [100].

### Long-term safety data for infliximab

The benefit:risk profile should be considered when selecting patients for infliximab therapy. The safety profile for infliximab is well established, and the labelling explains all risks (the following excerpts address TB, hepatitis and pregnancy):

Before starting treatment, all patients must be evaluated for active and inactive (‘latent’) tuberculosis [TB] according to local standards. In case of latent (or active) TB appropriate prophylactic (or therapeutic) measures have to be taken. Reactivation of hepatitis B has occurred in patients receiving a TNF-inhibitor including infliximab, who are chronic carriers of this virus. Some cases have had fatal outcome. Risk for HBV [hepatitis B virus] infection has to be evaluated before initiating Remicade therapy. Carriers of HBV who require treatment with Remicade need to be closely monitored for signs and symptoms of active HBV infection throughout therapy and for several months following termination of therapy. Effective anti-viral therapy may be needed. Post-marketing reports from approximately 300 pregnancies exposed to infliximab, did not indicate unexpected effects on pregnancy out come.

Due to its inhibition of TNFα, infliximab administered during pregnancy could affect normal immune responses in the newborn. […] Since the available clinical experience is too limited to exclude a risk, infliximab should not be administered during pregnancy.

*Remicade Summary of Product Characteristics*, *2009*[*89*]

In RA, a meta-analysis of seven randomised controlled trials (*n* = 2,100 patients) of duration ≤1 year in patients receiving either infliximab plus MTX or placebo plus MTX demonstrated that between-group differences for SAEs, serious infections, malignancy or death were not significant. The between-group difference for infections was close to significance (*P* = 0.06) [104]. The infliximab group had significantly more infusion reactions than the placebo group (*P* = 0.02). The number of withdrawals due to adverse events was also significantly higher in the infliximab group compared with the control group (*P* = 0.001). A network meta-analysis of six Cochrane reviews, all of which were updated to 2009 (31 randomised controlled trials, *n* = 17,668), confirmed that efficacy is similar among the TNF inhibitors [105]. Adverse reactions are thought to be related to TNF blockade, and to represent class effects of these agents [106].

In AS, a recent head-to-head, 2-year trial of infliximab and etanercept in 50 patients with late disease (mean 15.4 and 15.7 years, respectively) found that adverse events were mostly mild to moderate in both groups. There were no discontinuations for safety reasons and no opportunistic infections, TB, congestive heart disease, demyelinating disorders, lupus-like syndrome or malignancy [107]. In an open-label, 5-year (except for a short discontinuation at 3 years) randomised controlled trial of 69 patients with AS who received either infliximab or placebo, most early adverse events were mild to moderate, except one case of TB and one case of allergic bronchiocentric granulomatosis at 1 year [108]. At 3 years (*n* = 43), none of the six SAEs were considered causally related to infliximab [73]. At 5 years (*n* = 38) there were no safety concerns, and about one-half of the initial patient cohort was still being successfully treated [74]. These safety results are consistent with data from a large registry [94].

In PsA, the IMPACT 1 and IMPACT 2 studies demonstrated that infliximab was generally well tolerated. In the IMPACT 1 study (*n* = 104), the treatment groups had a similar incidence of all adverse events, treatment-related adverse events, infusion-related adverse events and both SAEs and severe adverse events during the placebo-controlled phase (weeks 0 to 16) and the crossover phase (weeks 16 to 50) [82]. In the IMPACT 2 study (*n* = 200), 67 out of 100 infliximab patients (67%) experienced an adverse event through week 24 (prior to crossover) and 147 out of 173 combined-infliximab patients (85%) experienced an adverse event through week 54 [84]. Through week 54, 22 out of 173 patients (12.5%) in the combined group also experienced an SAE. Importantly, adverse event incidence in the combined-infliximab group was similar between patients receiving MTX (87.5%) and patients not receiving MTX (82.5%) at baseline. When balanced with the improvement in signs and symptoms of PsA, QoL and physical function, and with the high degree of ACR and PASI response through 1 year of infliximab treatment, the authors concluded that the beneft:risk ratio was positive.

## Benefit:risk profile

Determining the benefit:risk profile of TNF inhibitors can be challenging, for reasons that include the lack of head-to-head clinical trials between drugs and the wide variability in the reported rates of SAEs by different studies. Infliximab, the drug of focus in the present review, has demonstrated efficacy in all rheumatological conditions (RA, AS and PsA) as well as other inflammatory disorders (Crohn’s disease, ulcerative colitis and psoriasis), and no new or unexpected safety signals have arisen over the years. Potential risks exist, but infliximab is generally well tolerated when clinicians appropriately select patients and adhere to indications and contraindications. Vigilance regarding important safety considerations continues to be necessary, as is the need for adequate patient screening and monitoring.

## Few questions remain

Research since 1990 has revealed that an immune-mediated inflammatory mechanism leading to the activation of proinflammatory cytokines underlies RA, AS and PsA. This knowledge has driven the development of anti-TNF agents. Today, TNF inhibitors effectively suppress and control the inflammation that drives these diseases. Suppression and control are critical to the prevention of irreversible tissue damage and disability. TNF inhibitors have therefore radically changed the entire therapeutic approach, which has shifted from mitigation of symptoms to blockade of progression.

As with any drug, patient response varies. A proportion of patients do not respond, insufficiently respond or lose an initial good response to classic TNF inhibitors. In such patients, other TNF inhibitors, including golimumab [109], or other agents, such as the B-cell-depleting chimeric antibody rituximab [110-112], the T-cell co-stimulation inhibitor abatacept [113], or the IL-6 receptor inhibitor tocilizumab [114], may be effective.

Studies of the TNF inhibitor infliximab stimulated most of the developments recognised today as pertinent for TNF inhibitors and also set the stage for other biologic agents. The first randomised controlled study in a rheumatic disease reported the efficacy of a single infusion of infliximab in RA patients 16 years ago [30]. Over the 12 years that followed licensing of the first TNF inhibitor for an inflammatory disease, research has shown that, for most patients, infliximab effectively treats signs and symptoms, provides rapid and prolonged suppression of inflammation and may prevent long-term disease progression in RA, AS and PsA. In RA, infliximab, like other TNF blockers, is highly effective for both early and established disease, and can induce clinical remission. Importantly, initial analysis shows that infliximab can even maintain remission for approximately 1 year drug free in patients with early RA. In AS, infliximab induces a rapid reduction in disease activity; and in PsA, infliximab treats not only joint symptoms, but also extra-articular manifestations, including skin disorders, dactylitis, enthesitis and nail disease.

More recently, as we have learned that some patients with RA experience RPD despite MTX therapy, an aggressive approach early in the disease course has been tried. Data are not yet widely available, but subset analyses have demonstrated reductions in potential markers of RPD (for example, CRP levels, ESR, swollen joint count, rheumatoid factor levels) and improved long-term benefits with the early addition of infliximab. Infliximab has been shown to halt joint destruction even in these patients, and predicting RPD may allow tailoring of biologic therapy in the disease course.

## Abbreviations

ACR, American College of Rheumatology; AS, ankylosing spondylitis; ASAS, Assessment of the SpondyloArthritis International Society; ASPIRE, Active-Controlled Study of Patients Receiving Infliximab for the Treatment of RA of Early Onset; ASSERT, Ankylosing Spondylitis Study for Evaluation of Recombinant Infliximab Therapy; ATTRACT, Anti-Tumor Necrosis Factor Trial in Rheumatoid Arthritis with Concomitant Therapy; BASDAI, Bath Ankylosing Spondylitis Disease Activity Index; BeST, Behandel Strategieën; CRP, C-reactive protein; DAS28, Disease Activity Score in 28 joints; DMARD, disease-modifying anti-rheumatic drug; ESR, erythrocyte sedimentation rate; EXPRESS, European Infliximab for Psoriasis (Remicade) Efficacy and Safety Study; GUEPARD, GUerir la PolyArthrite Rhumatoide Debutante [cure early RA]; IL, interleukin; IMPACT, Infliximab Multinational Psoriatic Arthritis Controlled Trial; MRI, magnetic resonance imaging; MTX, methotrexate; PASI, Psoriasis Area and Severity Index; PsA, psoriatic arthritis; RA, rheumatoid arthritis; RADIUS, Rheumatoid Arthritis DMARD Intervention and Utilization Study; RESPOND, Remicade Study in Psoriatic Arthritis Patients of Methotrexate-Naïve Disease; RPD, rapidly progressing disease; QoL, quality of life; SAE, serious adverse event; TB, tuberculosis; TNF, tumour necrosis factor.

## Competing interests

JSS has been a speaker for, and has received research grants from, Centocor, Inc., Schering-Plough Corporation (now Merck & Company, Inc.), Wyeth-Pfizer, and Merck & Company, Inc. PE has received consulting fees, lecture fees, and research grants from BMS, and has provided expert advice and undertaken clinical trials for Abbott, BMS, GSK, MSD, Roche, Schering, UCB, and Wyeth.
